# Metabolically Healthy Obesity

**DOI:** 10.1210/endrev/bnaa004

**Published:** 2020-03-04

**Authors:** Matthias Blüher

**Affiliations:** Medical Department III – Endocrinology, Nephrology, Rheumatology, University of Leipzig, Leipzig, Germany and Helmholtz Institute for Metabolic, Obesity and Vascular Research, Helmholtz Zentrum München, University Hospital Leipzig, Leipzig, Germany

## Abstract

Obesity contributes to reduced life expectancy, impaired quality of life, and disabilities, mainly in those individuals who develop cardiovascular diseases, type 2 diabetes, osteoarthritis, and cancer. However, there is a large variation in the individual risk to developing obesity-associated comorbid diseases that cannot simply be explained by the extent of adiposity. Observations that a proportion of individuals with obesity have a significantly lower risk for cardiometabolic abnormalities led to the concept of metabolically healthy obesity (MHO). Although there is no clear definition, normal glucose and lipid metabolism parameters—in addition to the absence of hypertension—usually serve as criteria to diagnose MHO. Biological mechanisms underlying MHO lower amounts of ectopic fat (visceral and liver), and higher leg fat deposition, expandability of subcutaneous adipose tissue, preserved insulin sensitivity, and beta-cell function as well as better cardiorespiratory fitness compared to unhealthy obesity.

Whereas the absence of metabolic abnormalities may reduce the risk of type 2 diabetes and cardiovascular diseases in metabolically healthy individuals compared to unhealthy individuals with obesity, it is still higher in comparison with healthy lean individuals. In addition, MHO seems to be a transient phenotype further justifying therapeutic weight loss attempts—even in this subgroup—which might not benefit from reducing body weight to the same extent as patients with unhealthy obesity. Metabolically healthy obesity represents a model to study mechanisms linking obesity to cardiometabolic complications. Metabolically healthy obesity should not be considered a safe condition, which does not require obesity treatment, but may guide decision-making for a personalized and risk-stratified obesity treatment.

## Graphical Abstract

**Graphical Abstract:**
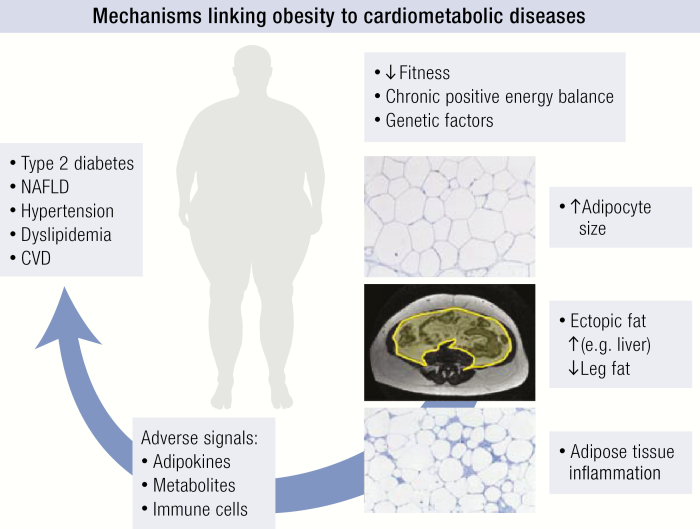

**Essential Points**

- Metabolically healthy obesity (MHO) is a concept derived from clinical observations that a subgroup of people with obesity do not exhibit overt cardiometabolic abnormalities.
- Although there is no standardized definition of MHO, the following criteria have been proposed in addition to the diagnosis of obesity (BMI ≥30 kg/m^2^): fasted serum triglycerides ≤1.7 mmol/l (≤150 mg/dl); HDL cholesterol serum concentrations >1.0 (>40 mg/dl) (in men) or >1.3 mmol/l (>50 mg/dl) (in women); systolic blood pressure (SBP) ≤130 mmHg; diastolic blood pressure ≤85 mmHg; fasting blood glucose ≤ 6.1 mmol/l (≤100 mg/dl); no drug treatment for dyslipidemia, diabetes, or hypertension; and no cardiovascular disease manifestation.
- With an age- and gender-dependent prevalence between ~10% to 30%, MHO is not a rare condition.
- Individuals with MHO are characterized by lower liver and visceral fat, but higher subcutaneous leg fat content, greater cardiorespiratory fitness and physical activity, insulin sensitivity, lower levels of inflammatory markers, and normal adipose tissue function compared to patients with metabolically unhealthy obesity (MUO).
- Metabolically healthy obesity most likely represents a transient phenotype, and individuals with MHO still have an indication for weight-loss interventions because their risk of developing cardiometabolic diseases may be lower compared to MUO, but it is still higher than in metabolically healthy lean people.

Since the 1970s, global obesity prevalence has nearly tripled in adults and has risen even more dramatically in children and adolescents (1–3). Obesity contributes to a reduced life expectancy of up to ~20 years due to increased mortality from noncommunicable diseases, including atherosclerotic cardiovascular diseases, type 2 diabetes, and certain types of cancer (4–7). In addition to the consequences of obesity at the individual level, the obesity pandemic may create an enormous health burden for society (8).

According to the World Health Organization (WHO), obesity is defined as “abnormal or excessive fat accumulation that presents a risk to health” (9). In contrast to the view that obesity only represents a risk factor for diseases, the World Obesity Federation declared obesity itself as a chronic, relapsing progressive disease (10). This has been justified by an epidemiological-model approach that considers the pathophysiology of obesity, an interaction of environmental factors (availability and accessibility of energy-rich food, low requirements for physical activity), with genetic susceptibility, resulting in a positive energy balance and higher body weight (10). The strong mechanisms promoting weight gain and defending a higher body weight even against targeted weight-loss interventions further argue to the view that obesity is a disease rather than a decision (3, 11). However, it has been found surprisingly difficult to define what a disease is (12). If a disease were simply the opposite of health, the concept of “healthy obesity” (and the topic of this review article) would be a contradiction in terms. The term “healthy obesity” is an illustration of the notion that health is context-dependent, and whether people consider themselves ill depends on a variety of factors (12). In addition, the definition of a disease may change over time as a result of health expectations, due to improving diagnostic tools, and for other social and economic reasons (12). In this context, the definition of obesity as a disease would have a strong impact both on the individual (stigmatization, self-esteem) and the society (attention by healthcare professionals or politicians) (13). It could affect decisions, how limited healthcare resources are allocated, and how to position obesity within the context of investments for the treatment of obesity-related diseases. 

One pragmatic approach to reduce the medical and socioeconomic costs associated with obesity treatment could be to prioritize those patients who will benefit the most from weight-loss interventions. Such risk-stratified obesity treatment would require better tools to measure obesity-related morbidity and mortality risk. In many current obesity treatment guidelines, diagnosis of obesity and treatment decisions are based on a body mass index (BMI) ≥30 kg/m^2^ (14–17) despite the inability of BMI to accurately predict cardiometabolic risk or to define total and central abdominal fat mass (11, 18). At any given BMI, the variation in comorbidities and health risk factors is remarkably high (18). Observational data from independent studies show that a subgroup of individuals with obesity may be protected from obesity-related cardiometabolic diseases or may be at a significantly lower risk than estimated from the positive association between BMI and cardiometabolic risk (19). This subphenotype has been described as MHO and is characterized by the absence of cardiometabolic abnormalities, including insulin resistance, impaired glucose tolerance, dyslipidemia, and hypertension despite excessive body fat accumulation (19–27). This short review focuses on the biological mechanisms underlying MHO and discusses whether the concept of MHO may have clinical implications for the prediction of cardiometabolic diseases and for stratified obesity treatment decisions. The review is based on a systematic PubMed search and prioritized more recent original articles as well as reviews. Searching the term “obesity” yields more than 320 000 citations, the term “obesity and metabolic diseases” more than 84 500 citations as of November 2019, whereas the search term “metabolically healthy obesity” yields 1080 citations. From the literature search strategy it became clear that the concept of MHO was attracting more attention since the early 2010s, now with ~160 citations per year.

## Concept of Metabolically Healthy Obesity

The concept of metabolically healthy obesity developed from Jean Vague´s observations in the 1950s that individuals with obesity have a different predisposition to diabetes and atherosclerosis, which could be related to body fat distribution (28). Since then, MHO has been described in clinical observations and epidemiological, prospective cohort, and intervention studies (19–27, 29, 30). It is now well established that there are people with obesity who do not exhibit metabolic and cardiovascular complications at a given point in time (29–34). However, it could be debated whether MHO represents a distinct and stable phenotype and whether MHO has clinical relevance for the prediction of type 2 diabetes and cardiovascular disease risk. The concept of MHO may serve as a model to better understand the mechanisms linking obesity to cardiometabolic diseases.

### Definition of MHO

Importantly, there is no unified definition of MHO (31–34). Despite the general consensus that a BMI ≥30 kg/m^2^ is a prerequisite for the definition of MHO, more than 30 different definitions of metabolic heath are used in clinical studies (33). Metabolically healthy obesity has been frequently defined by the absence of any metabolic disorder and cardiovascular disease, including type 2 diabetes, dyslipidemia, hypertension, and atherosclerotic cardiovascular disease (ASCVD) in a person with obesity (Table 1) (31–35). However, there is a large variation between investigators with regard to the MHO classification criteria and specific cutoff values for each parameter (Table 1) even to an extent that some cardiometabolic abnormalities were accepted in the category of MHO (22, 31–33, 35–38). The heterogeneous MHO definitions represent an important limitation for the interpretation of studies reporting a wide range of associations between MHO, cardiovascular disease, mortality, and the risk for metabolic diseases (27, 34, 39, 40). In addition, differences in diagnostic criteria may define MHO subpopulations, which only have little overlap in key cardiometabolic parameters (24). As an example, more than 40% of participants in the National Health and Nutrition Examination Survey (NHANES) III program were classified as MHO using the National Cholesterol Education Program (NCEP) Adult Treatment Panel III (ATP III) criteria for metabolic syndrome (41), but only 20% fell into the MHO category using more strict insulin sensitivity parameter cutoffs (42). These uncertainties in defining MHO may imply that MHO does not represent a distinct biologically determined subgroup of individuals with obesity. More recent data suggesting that the MHO phenotype is not a cardiometabolically benign condition (43) seem to justify the argument that MHO has very limited relevance as a public health target and should not be treated differently from obesity with established type 2 diabetes and/or cardiovascular diseases (CVD) (32, 44, 45).

**Table 1.** Proposed criteria for harmonized definitions of metabolically healthy obesity in adults.

	BioSHaRE-EU Healthy Obese Project (31)		Lavie et al (35)
**Obesity Classification**	**BMI ≥ 30kg/m** ^**2**^ **Plus All of the Criteria**		**BMI ≥ 30kg/m** ^**2**^ **Plus 1 to 4 of the Criteria**
	**Less Strict Criteria**	**Strict Criteria**	-
Blood pressure	≤ 140 mmHg	≤ 130 mmHg	≤ 130 mmHg
Systolic blood pressure	-	≤ 85 mmHg	≤ 85 mmHg
Diastolic blood pressure	≤ 90 mmHg	-	-
	No antihypertensive drug treatment		
Blood glucose	≤ 7.0 mmol/l	≤ 6.1 mmol/l	≤ 5.6 mmol/l
	No blood glucose-lowering medication or diagnosis of type 2 diabetes		
Fasting triglycerides	≤ 1.7mmol/l	≤ 1.7mmol/l	≤ 1.7mmol/l
Non-fasted state	≤ 2.1mmol/l	≤ 2.1mmol/l	-
	No drug treatment for elevated triglycerides		
HDL-cholesterol	>1.03 mmol/l (men) >1.3 mmol/l (women)	>1.03 mmol/l (men) >1.3 mmol/l (women)	>1.0 mmol/l (men) >1.3 mmol/l (women)
	No drug treatment for reduced HDL-cholesterol		
Diagnosis of CVD	No	No	-

Adapted from references (31, 35).

The need for standardized MHO criteria has been recently addressed by the BioShare-EU project (31) and by Lavie and colleagues (35). In the context of the Healthy Obese Project, the data of 10 population-based cohort studies from 7 countries (Estonia, Finland, Germany, Italy, Netherlands, Norway, and the UK), including more than 163 000 adults (of whom 17% had obesity [11 465 men and 16 612 women]) aged between 18 and 80 years were evaluated to compare key characteristics to define MHO by clinical and metabolic factors (31) (Table 1). The collaborators distinguished 2 levels of strictness for the MHO definition (Table 1). More recently, a harmonized definition of MHO in adults has been proposed based on the diagnosis of obesity (BMI ≥30 kg/m^2^) and meeting all of the criteria: serum triglycerides ≤1.7 mmol/l (≤150 mg/dl), HDL-cholesterol serum concentrations >1.0 (>40 mg/dl) (in men) or >1.3 mmol/l (>50 mg/dl) (in women), systolic blood pressure (SBP) ≤130 mmHg, diastolic blood pressure ≤85 mmHg, no antihypertensive treatment as an alternative indicator, fasting blood glucose ≤ 5.6 mmol/l (≤100 mg/dl), and no drug treatment with glucose lowering agents (35) (Table 1). These definitions of MHO seem to be more practicable compared to previous attempts to define MHO using parameters for insulin sensitivity (eg, euglycemic-hyperinsulinemic clamps, HOMA-IR, Matsuda-index) or systemic inflammation (eg, C-reactive protein) (reviewed in 25). In contrast to the origins of the MHO concept (which may have included patients with hypertension or type 2 diabetes), more recent definitions (31, 35) exclude individuals who meet only1 of the metabolic syndrome criteria.

Importantly, the concept of MHO can only be applied to individuals fulfilling the described cardiometabolic criteria and should not be misinterpreted as a subgroup of people with obesity without any health impairments (32). In addition to metabolic diseases (eg, type 2 diabetes, dyslipidemia, fatty liver disease) and cardiovascular diseases (eg, hypertension, myocardial infarction, stroke), obesity is associated with osteoarthritis, back pain, asthma, depression, cognitive impairment, and some types of cancer (eg, breast, ovarian, prostate, liver, kidney, colon)—all of which can have an impact on reduced quality of life, unemployment, lower productivity, and social disadvantages (5, 7, 9, 10, 18, 30). Therefore, the diagnosis of “obesity” should remain an indication to initiate treatment—even in those individuals without any cardiometabolic abnormalities at the time of diagnosis.

### MHO prevalence

Assumptions about the prevalence of MHO are not very reliable and show a large variation due to a lack of standardized definitions of this phenotype (32, 46). Depending on which MHO definitions are used, prevalence of MHO has been shown to range between 4.2% and 13.6% in a random sample from a Chinese adult population (46). A recent meta-analysis from 12 cohort and 7 intervention studies found a 35% prevalence of MHO with significant regional differences (47). In general, MHO seems to be more prevalent in women than in men and decreases with age (31). Great regional and gender-related variations in MHO prevalence has been found in the BioSHaRE-EU Healthy Obese Project, which estimated the age-standardized prevalence of MHO at ~12% across all cohorts (31). In their analysis of 10 independent cohorts from different European countries, the prevalence of MHO varied in women from 7% in the Finnish Health 2000 study to 28% in the United Kingdom National Child Development Study (NCDS) birth cohort, and in men from 2% in Finland DIetary, Lifestyle and Genetic factors in the development of Obesity and Metabolic syndrome (DILGOM) to 19% in the Collaborative Health Research in South Tyrol Study (CHRIS) from Italy (31). The greatest gender difference has been found in the NCDS, with a MHO prevalence of 9% in men compared to 28.4% in women, whereas MHO prevalence was similar in men (19%) and women (21.1%) in a cohort from Italy (31). Importantly, MHO prevalence estimates can only be compared across different cohorts or studies if the same criteria to define MHO are applied. As an example, the 68% MHO prevalence observed in a large recent study of 3.5 million men and women for which validated electronic health records were available in the context of The Health Improvement Network (THIN) database is most likely overestimated, because the definition of MHO did not consider blood glucose, blood pressure, or lipid parameter cutoffs (43, 44). Metabolically healthy obesity has also been found in Asian and African populations (depending on diagnostic criteria and based on a BMI ≥25 kg/m^2^ cutoff for obesity), ranging from 4.2% in a cohort from China with an obesity prevalence of 24.3% (46) to 13.3% among Asian Indians with a 28.1% obesity prevalence (48) and 28.5% in African Americans (49). Among 1054 Hispanic American participants of the Insulin Resistance Atherosclerosis Study (IRAS), 19% were categorized as MHO (50). Data from the NHANES III program suggest an MHO prevalence of ~17% in Americans with European or African ancestry (42).

In children and adolescents, MHO may be a more frequently observed condition. In a cross-sectional study from Canada, which included girls and boys ages 8–17 with a BMI ≥ 85^th^ percentile, prevalence of MHO was 21.5% when cardiometabolic risk factors (blood pressure, serum lipids, glucose) were considered and 31.5% if insulin resistance parameters were applied to define MHO (51). In children and adolescents of the Korea National Health and Nutrition Examination Survey, MHO prevalence was between 36.8% (for a cardiometabolic risk factor-based definition) and 68.8% (for insulin resistance criteria) (52). Irrespective of the definitions used and the remarkable regional and gender variation, MHO does not appear to be a rare condition (35).

## Biological Mechanisms Underlying Metabolically Healthy Obesity

**Figure 1.**
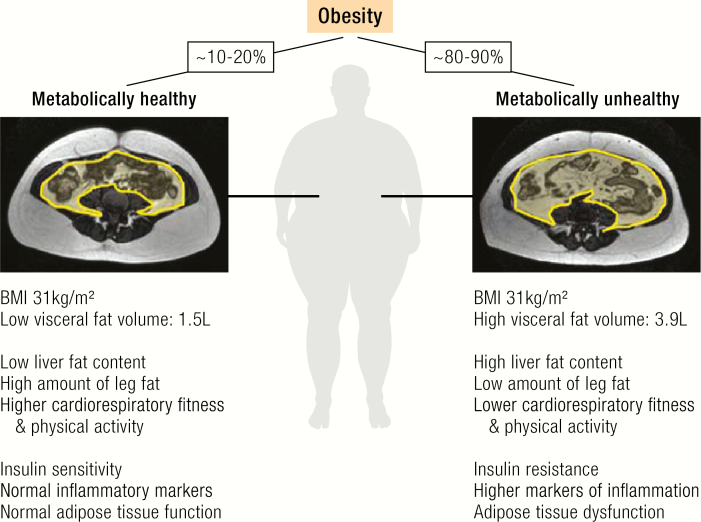
Phenotypic traits associated with metabolically healthy versus unhealthy obesity. Individuals with metabolically healthy obesity (MHO, prevalence ~10–30%) are characterized by lower liver and visceral fat mass, higher leg fat content, greater cardiorespiratory fitness and physical activity, insulin sensitivity, normal inflammation markers, and preserved adipose tissue function compared to patients with metabolically unhealthy obesity (MUO, prevalence ~80–90%). Transabdominal MRI scans with highlighted (yellow) visceral fat depot area from 2 women with the same age and BMI, but either MHO or MUO show ~2.6-fold higher visceral fat deposition associated with MUO (pictures provided by Nicolas Linder).

**Figure 2.**
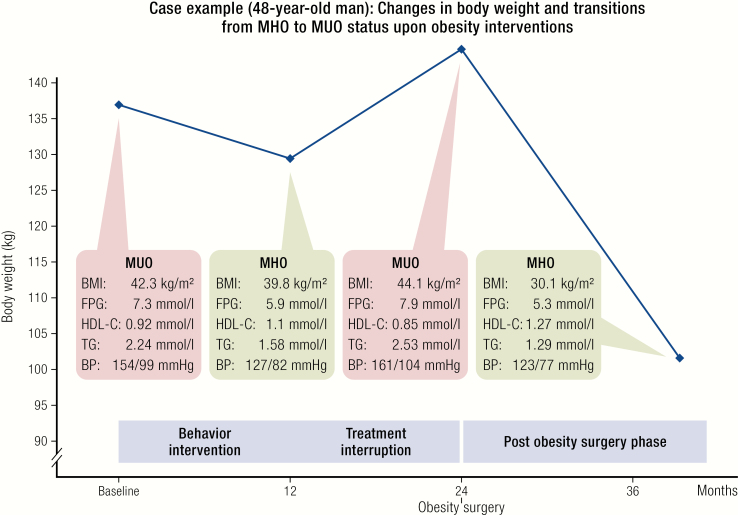
Metabolically healthy obesity is a transient phenotype. Case example for a 48-year-old man undergoing different weight-loss interventions. At baseline, the patient presented with MUO as defined by reference (31). After 12 months of a behavior intervention program (calorie restricted diet, increased physical activity, and psychosocial support), the phenotype changed into MHO. Because treatment was not continued for the subsequent 12 months, there was a weight regain associated with a phenotype transition to MUO. At 24 months, the patient underwent a laparoscopic Roux-en-Y gastric bypass surgery, which resulted in significant weight loss and improvements in all criteria defining MHO. The case demonstrates that transitions between MUO and MHO are not unidirectional and may change over time, for instance in response to weight-loss interventions. Abbreviations: BMI, body mass index; BP, blood pressure; FPG, fasting plasma glucose; HDL-C, high-density lipoprotein cholesterol; TG, triglycerides.

Despite the debate about the clinical implications of MHO as a “diagnosis” (20, 21, 32, 44, 45), obesity without cardiometabolic abnormalities provides a unique human model system to study mechanisms linking the factors that promote weight gain and fat accumulation to obesity-related cardiometabolic complications. Over the past years, a number of biological mechanisms and phenotypic characteristics have been identified that differentiate individuals with MHO from those with metabolically unhealthy obesity (MUO) (Fig. 1). In a large BMI-stratified cohort, Stefan et al (20, 23) linked high liver fat content and predominantly abdominal (including visceral) adiposity to MUO, whereas greater insulin sensitivity, better insulin secretion, cardiorespiratory fitness, and lower body subcutaneous fat mass were associated with an MHO phenotype. Admittedly, these associations do not solve the question of whether and which phenotypic traits may cause or only reflect a protection against cardiometabolic abnormalities in MHO. Importantly, the biological correlates of MHO were similarly associated with metabolic health across the BMI range from lean to overweight to obese (23). In this context, it has been recently shown that higher trunk fat in normal weight postmenopausal women is associated with increased ASCVD incidence, whereas higher leg fat predicted lower ASCVD risk (53). These data further support the notion that altered and ectopic (eg, liver, visceral fat depots, skeletal muscle) fat distribution is a stronger determinant of metabolic health as increased fat mass itself (23–26). Beyond the associations of BMI, hepatic steatosis has been shown to predict the risk of developing type 2 diabetes (54) and ASCVD (55, 56). Altered fat distribution with increased visceral and liver fat deposition and low leg fat mass might be the result of an impaired expandability of healthy subcutaneous adipose tissue stores (57–59). In analogy to human lipodystrophy, MUO might be the result of an inability of subcutaneous adipose tissue to further expand upon a chronic positive energy balance. Impaired adipose tissue function might indeed mechanistically link long-term energy imbalance between too many calories consumed and too few calories expended and end organ damage, including hepatic steatosis, type 2 diabetes, and ASCVD (Fig. 2).

To further elucidate the potential role of adipose tissue function in defining metabolic health despite obesity, we studied pairs of individuals with MHO, which have been matched for age, gender, and BMI, but were either insulin sensitive or resistant in euglycemic-hyperinsulinemic clamps (22). In addition to higher visceral and liver fat amounts in insulin resistant obesity, we found insulin sensitive MHO to be associated with less immune cell infiltration into visceral fat depots, lower mean adipocyte size, and a favorable adipokine secretion pattern (22). In contrast, a proinflammatory, diabetogenic and atherogenic secretion pattern may contribute to the development of MUO. Our data supports models for the development of MUO (60, 61) in which ectopic fat and impaired adipose tissue function may lead to systemic insulin resistance, lipotoxicity, and a proinflammatory state and could, therefore, play a causal role in the transition from MHO to MUO (Fig. 2).

Moreover, we found a distinct pattern of circulating signaling molecules associated with MHO (22). Individuals with insulin sensitive MHO are characterized by higher adiponectin and neuregulin 4 (62) and lower C-reactive protein (CrP), progranulin, chemerin, fetuin-A, retinol binding protein-4 (RBP4) (22), dipeptidyl peptidase-4 (DPP4) (63), and serum concentrations compared to individuals with insulin resistant obesity (64). Interestingly, MHO could be best predicted on the basis of macrophage infiltration into visceral adipose tissue and adiponectin serum concentrations (22). Signals from adipose tissue may include peptide hormones (adipokines), immune cells, and metabolites, which either specifically or as a pattern contribute to the development of type 2 diabetes, fatty liver disease, endothelial dysfunction, and cardiovascular diseases (57, 60, 64). In a recent unbiased cluster analysis of 12 signaling molecules, adiponectin, adipocyte fatty acid-binding protein (AFABP), chemerin, and fibroblast growth factor (FGF) 21 showed the strongest associations with parameters of metabolic health (65). However, it remains an open question for prospective epidemiological studies whether circulating parameters can predict conversions from MHO to MUO. Altered signaling molecule signatures may either directly affect target tissue via receptor mediated mechanisms (eg, leptin’s effects on satiety regulation in the brain) or contribute indirectly (eg, modulation of insulin secretion through free fatty release from visceral fat depots) to increasing cardiometabolic diseases (57).

The importance of adipose tissue function in the determination of the obesity subphenotype is further supported by data from transgenic animal studies. For example, mice with a transgenic overexpression of the insulin-sensitizing adipokine adiponectin or the mitochondrial protein mitoNEET—both on the background of leptin-deficient *ob/ob* mice—resemble the human MHO phenotype with preserved insulin sensitivity and low liver and muscle fat despite extreme obesity (66, 67).

Increasing physical activity and the preservation of cardiorespiratory fitness are well established interventions to reduce the obesity-related risk for type 2 diabetes and ASCVD (68). Both in children and adults, higher physical activity and cardiorespiratory fitness have been recognized as an important correlate of the MHO phenotype (51, 69, 70). Importantly, higher fitness levels in MHO compared to MUO may also be an indicator for a healthier lifestyle and does not exclude other behavior factors underlying MHO.

## Transitions between Metabolically Healthy and Unhealthy Obesity

**Figure 3.**
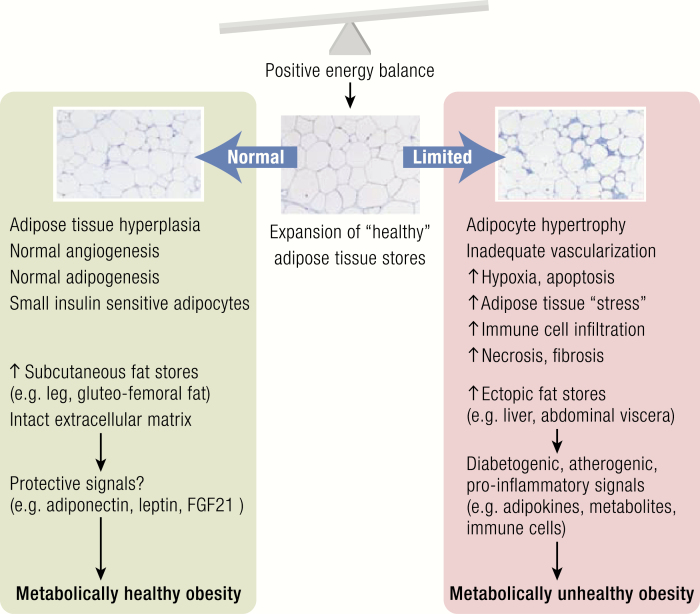
Adipose tissue dysfunction and development of metabolically unhealthy obesity. A chronically positive energy balance requires expansion of adipose tissue (AT) to store excess energy. Adipose tissue responds to higher storage demands by increasing the adipocyte number through adipogenesis from precursor cells (hyperplasia) and through adipocyte hypertrophy. If expansion of healthy fat stores (eg, subcutaneous leg fat) and the ability of AT to respond to excess calorie intake with (“healthier”) hyperplasia are impaired, AT dysfunction may develop, which is characterized by ectopic fat deposition (eg, liver, abdominal visceral depots, skeletal muscle, pancreas) and a sequence from adipocyte hypertrophy, hypoxia, inadequate vascularization, AT stress, and immune cell infiltration, apoptosis, and increased production of profibrotic extracellular matrix proteins contributing to fibrosis. Adipose tissue dysfunction leads to the release of proinflammatory, diabetogenic, and atherogenic signals (eg, adipokines, fatty acids from increased lipolysis, other metabolites, immune cells), which may contribute to end organ damage (eg, liver, skeletal muscle, pancreas, vasculature) and the development of metabolically unhealthy obesity. In contrast, healthy expansion of AT leads to metabolically healthy obesity through an increased AT storage capacity (serving as a safe “metabolic sink”) and the secretion of a beneficial adipokine profile (eg, adiponectin, FGF-21, leptin) (adapted from references (60, 61).

Obesity has been considered a chronic relapsing and progressive disease (10, 71), a definition which is most likely also applicable to MHO. Indeed, individuals in long-term obesity treatment programs may undergo cycles of weight loss and weight regain accompanied by their phenotype changing from MUO to MHO and back to MUO (Fig. 3). Such transitions between metabolic status are not specific to obesity and have also been identified in children and adolescents (72). Moreover, almost 50% of the Multi-Ethnic Study of Atherosclerosis (MESA) participants, which have been defined as MHO at baseline, developed metabolic abnormalities during the ~12-year follow-up period (40). This finding is supported by a meta-analysis of 12 studies including more than 5900 individuals with 3–10-year follow-up, which demonstrates that almost half of the participants classified as MHO developed at least 1 metabolic abnormality (47). Individuals with MHO can be found at any age, but in groups with increasing age the prevalence of MHO has been shown to be consistently lower (31). The lower prevalence of MHO in postmenopausal compared to premenopausal women and a 30% transition from MHO to MUO over menopause (73) suggests that changes in sex hormones may play a role in the transition from MHO to MUO. Among participants of the prospective Pizarra study, ~30% of individuals diagnosed with MHO at baseline converted to MUO in the 6-year follow-up investigation (74). Importantly, the transition from MHO to MUO is not necessarily a one-way road, as individual interventions illustrate (Fig. 3). Moreover, data from 3743 women (51%) and men ≥ 18 years of age in the North West Adelaide Health Study show that conversion from MUO to MHO occurred without significant gender differences in 16% of the participants in up to 10-year recall visits (75). Persistence of MHO was related to a younger age, sustained lower waist circumference, more peripheral fat distribution in women, and favorable diabetes and cardiovascular disease outcomes (75). A recent analysis from the Clinical Practice Research Datalink (CPRD), a large-scale primary care database from the UK containing data of 231 399 patients with a recorded BMI of ≥35 kg/m^2^, suggested that men are more prone to transitions from MHO to MUO (76). Finally, 30-year follow-up data from 90 257 participants of the Nurses’ Health Study robustly confirmed the frequent transition from MHO to MUO and demonstrated a decline in metabolic health with age across the entire BMI range (27). During this long observation period, it could also be shown that there are individuals maintaining their MHO status over a long period, which did not translate into reduced CVD risk to the level of metabolically healthy lean participants. Taken together, longitudinal studies demonstrate that metabolic health is not a stable condition, does not only depend on the obesity status, and deteriorates with ageing. On the other hand, MUO may also be considered a temporary trait, which could be reversed into MHO by targeted interventions.

## Risk of Type 2 Diabetes and Cardiovascular Diseases in Metabolically Healthy Obesity

**Figure 4.**
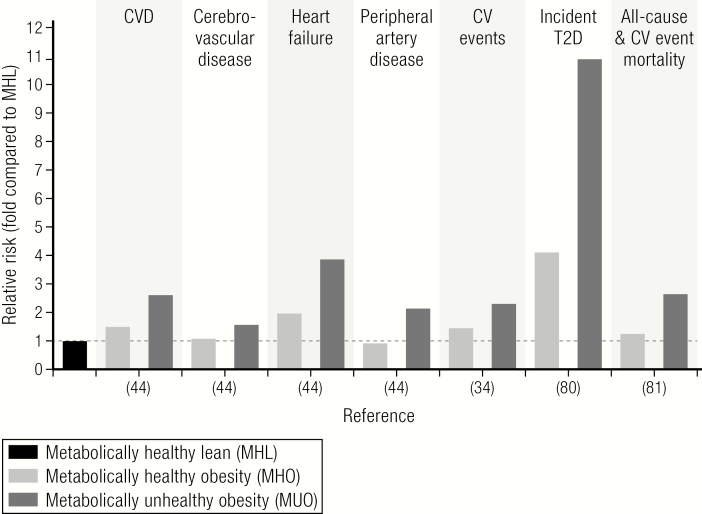
Risk of CVD and cardiovascular events, type 2 diabetes (T2D), and all-cause and/or CVD event mortality in MHO. Metabolically healthy lean (MHL) served as a reference group, and the mean relative risk for incident diseases, events, or mortality was compared between the MHO group (defined as absence of any metabolic abnormalities) and a group of individuals with MUO. Data are extracted from previous meta-analyses (34, 79, 80) or a large recent cohort study (43). For data from reference (44), only the subgroup of MUO with 3 metabolic comorbidities (= highest relative risk) is displayed despite evidence for gradually increasing risk (in all categories) with the increased number of metabolic abnormalities (ranging from 1–3).

Obesity significantly increases the risk of developing type 2 diabetes and cardiovascular diseases (6, 30, 34, 77–79) (Fig. 4). Because the increased cardiometabolic risk in people with obesity may be mediated by metabolic (elevated glucose, altered lipid profile) and cardiovascular (hypertension, circulating atherogenic factors) abnormalities, it has been postulated that people with MHO are protected against type 2 diabetes, ASCVD, and even all-cause mortality (20, 36, 78, 80). Indeed, MHO could be considered a “benign condition” because the meta-analyses of prospective studies consistently demonstrated that MHO is associated with a significantly lower incidence of type 2 diabetes and cardiovascular diseases (27, 81). However, the view that MHO is a benign subphenotype of obesity has been challenged by data from large epidemiological studies and meta-analyses demonstrating that individuals with MHO are at a higher risk for ASCVD, cerebrovascular disease, heart failure (43, 82, 83), cardiovascular events (34), type 2 diabetes (76), and all-cause mortality (78) in comparison to metabolically healthy lean individuals (Fig. 4). The only exception was a reduced risk of peripheral artery disease in MHO compared to metabolically healthy lean individuals (43). Noteworthy, data from meta-analyses demonstrating an increased cardiometabolic risk for MHO compared to healthy lean people do not exclude the possibility that in individual prospective trials MHO might not be associated with an increased risk of, for example, acute myocardial infarction compared to metabolically healthy lean individuals (39). There are indications that people with MHO may develop cardiometabolic complications of obesity with a delay compared to MUO (27), and one may speculate that in analogy to lower BMI-class obesity, people with MHO gain noncommunicable disease-free years (84). Interestingly, participants of the Nurses´ Health Study who maintained MHO over a long time still had a 57% higher risk of CVD than those women with a stable normal body weight (27). In the same study it has been shown that the CVD risk increased in women who converted from MHO to MUO compared to those with stable MHO (27). The increased CVD risk in those women converting from MHO to MUO was mainly driven by incident type 2 diabetes and hypertension (27). Data from more than 3.5 million individuals collected in THIN demonstrated that cardiometabolic risk increased from normal weight to overweight and obese, but was more pronounced with an increasing number of metabolic abnormalities (43). It remains open as to whether (and to which extent) fat accumulation itself (43) increased visceral and ectopic fat (18, 85) and/or whether the degree of respiratory fitness and physical activity (86) are the major contributors to these differences in cardiometabolic risk (35). Importantly, obesity significantly increases the risk of heart failure by adverse effects on cardiac structure and function by affecting systolic and diastolic ventricular function and as a result of ASCVD (87). There is strong evidence from epidemiological studies that obesity independently of other cardiometabolic risk factors, including high LDL-cholesterol, smoking, or diabetes, increases the risk for CVD (30, 34, 43, 82) (Fig. 4). Whereas the beneficial effects of behavioral and pharmacological weight-loss interventions on reducing the risk of developing type 2 diabetes has been well established (88–90), the evidence regarding cardiovascular health outcomes associated with weight loss is still limited (18, 85).

Collectively, there has been accumulating evidence over the past decades supporting the notion that obesity has long-term harmful consequences on cardiometabolic health even in those individuals with MHO (32, 35). Although MHO is associated with a substantially lower risk compared to MUO, it does not protect against cardiometabolic disease and should therefore not be treated as a benign condition (32, 45).

## Applying the Concept of Metabolically Healthy Obesity in Clinical Practice

Obesity treatment is challenging. First, conservative treatment strategies aiming at behavior changes have very little long-term success and the weight-loss effect of current behavior and pharmacological interventions is only in the range between 3–10%. Secondly, weight maintenance after weight loss is difficult to achieve. Finally, the most effective treatment, obesity surgery, is frequently not available and certainly not a solution for a health problem with the magnitude of the obesity pandemic. In the context of these challenges, the concept of MHO may have clinical implications with regard to treatment stratification and prioritization of those people who may gain the most from weight-loss interventions. The need to prioritize obesity treatment is most obvious for bariatric surgery, because the severity of obesity and its comorbid conditions as well as waiting time are associated with an increase in morbidity and mortality (90). Whether the concept of MHO may help to use typically limited treatment resources more effectively, to avoid unnecessary intense treatment programs with a low success rate (MHO as a contraindication for weight-loss interventions?) or whether it may delay an indicated obesity treatment is an open debate (24, 25, 32).

The increased risk of individuals with MHO for type 2 diabetes and CVD and the risk of transition into MUO clearly justify that obesity treatment is also indicated in people with MHO (32). One could even argue that individuals with MHO have a high treatment priority because they may benefit the most from preserving metabolic health. This suggestion is supported by data from bariatric surgery interventions showing that shorter duration of type 2 diabetes and better parameters of hyperglycemia are major determinants of diabetes remission and metabolic health (91). Moreover, women who maintained MHO during follow-up visits of the Nurses’ Health Study have a lower cardiovascular disease risk compared with metabolically healthy women who converted to an unhealthy phenotype (27). In contrast, short-term behavior interventions demonstrated that individuals with MHO may benefit less from obesity treatment compared to people with MUO (36, 92, 93). Importantly, treatment of obesity does not necessarily have to focus on weight loss, and improving health might be a better treatment target than the extent of weight loss. The Edmonton Obesity Staging System (EOSS) (94) therefore suggests an obesity classification based on clinical assessments of health and functional status. For an individual with MHO without functional impairment (EOSS stage 0), avoiding further weight gain would be recommended, but the health benefits of an aggressive weight-loss program are considered marginal (95). On the other hand, bariatric surgery interventions have been shown to be as effective in MHO compared to MUO patients with regard to cardiometabolic outcomes contradicting an obesity stratification based on the MHO status (35, 95–97). Moreover, weight loss extent-dependent improvements in health parameters and outcomes have been described, for example, in the Action for Health in Diabetes (Look AHEAD) trial (98) and seem to apply also to individuals with MHO (99, 100). A moderate weight loss of about 10% may be sufficient to change an obesity phenotype with cardiometabolic abnormalities into MHO (21, 69, 100).

At the moment, there are no randomized controlled obesity treatment trials comparing cardiometabolic outcomes between individuals with MHO and MUO, which would support any treatment stratification depending on the MHO status. Until such data are available, early treatment of obesity should also be recommended for individuals with MHO with the major aim to preserve cardiometabolic health and to prevent the natural course of MHO to convert into MUO with aging. From the public health point of view, individuals with MHO may have a lower priority for early access to treatment and more aggressive weight-loss strategies. Obesity treatment targets should shift from weight loss to health parameter goals. Maintaining favorable cardiometabolic health parameters could be easier to achieve and may require only moderate weight loss (Fig. 2) in individuals with MHO.

## Improving Cardiometabolic Health by Obesity Pharmacotherapies

For a person with obesity, it is very difficult to achieve and maintain a normal body weight with behavior interventions. Bariatric surgery is not always suitable, indicated, or wanted by the patients. Therefore, it seems to be more realistic to convert cardiometabolically unhealthy obesity into MHO. Significant health improvements can already be achieved by a moderate 3–10% weight loss (71, 101). If a clinically meaningful weight loss cannot be reached by a combination of energy deficit nutrition, increased physical activity, and behavior support, the next step of escalation would be adding pharmacotherapies for weight management (101–104). Currently, there are 5 medications approved for chronic weight management in the U.S., 3 of which have been approved in the European Union (101) (Table 2). These pharmacotherapies cause weight loss through different modes of action, with varying efficacy (Table 2) and specific side-effects, which both the prescriber and the patient should be aware of (101). A more detailed description of specific weight management medications would be beyond the scope of this short review and can be found elsewhere (101–103). In general, obesity pharmacotherapies should be used to reinforce patients to change eating behaviors and support nonpharmacological treatment strategies, but they may also contribute tothe improvement of several aspects of cardiometabolic health (Table 2). In addition to weight loss, the majority of obesity pharmacotherapies have been shown to improve at least some parameters defining metabolic health (Table 2). For example, orlistat treatment over 4 years (105) and liraglutide 3.0 mg treatment over 3 years (90) have been shown to reduce the risk of developing type 2 diabetes in people with prediabetes. Importantly, there is still an unmet need to develop more efficacious and safe pharmcotherapies against obesity. During the last ~10 years, a generation of molecules with agonism at the glucagon-like peptide-1 (GLP-1) receptor have emerged as promising tools in the pharmacotherapy of obesity (106–108). With liraglutide 3.0 mg and semaglutide there are GLP-1 receptor agonists, which are either already approved (liraglutide) or in clinical development (semaglutide), for weight management in obesity that demonstrated a cardiovascular benefit for patients with type 2 diabetes in the Liraglutide Effect and Action in Diabetes: Evaluation of Cardiovascular Outcome Results (LEADER) and Trial to Evaluate Cardiovascular and Other Long-term Outcomes With Semaglutide in Subjects With Type 2 Diabetes (SUSTAIN6) trials (109, 110). In a recent weight-loss intervention, doses of more than 0.2 mg of semaglutide demonstrated significantly superior weight-loss compared to liraglutide (111).

**Table 2.** Approved medications for weight management.

Medication (full dose & adminstration)	Main Mechanism of Action	Approval Status	Mean Weight Loss (% from Baseline)		Effects on MHO Diagnostic Parameters
			Placebo	Medication	
Orlistat (120 mg TID, oral)	Pancreatic lipase inhibitor	USA, EU	-2.6%	-6.1%	HbA1c lowering; lowers risk of developing type 2 diabetes in individuals with prediabetes; HDL-C decrease; lowers BP; LDL-C lowering
Phentermine (15–30 mg, QD, oral)	Sympatho-mimetic	USA, only for short-term use	No data available for monotherapy treatment of ≥52 weeks		
Lorcaserin (10 mg, BID, oral)	5-HT_ac_ serotonin agonist	USA	-2.5%	-5.8%	HbA1c lowering; HDL-C increase; lowers BP
Phentermine/topiramate ER (titration) (15 mg/92 mg, QD, oral)	Sympatho-mimetic/ anticonvulsant	USA	-1.2%	-7.8% to -9.8% (dose dependent)	HbA1c lowering; HDL-C increase; lowers BP
Naltrexone SR/ bupropion SR (titration) (32 mg/360 mg, BID, oral)	Opiod receptor antagonist/ dopamine and noradrenaline reuptake inhibitor	USA, EU	-1.3%	-5.4%	HbA1c lowering; BP increase; HDL-C increase
Liraglutid (titration) (3.0 mg, QD, subcutaneous injection)	GLP-1 receptor agonist	USA, EU	-3.0%	-7.4%	HbA1c lowering; lowers risk of developing type 2 diabetes in individuals with prediabetes; lowers BP; HDL-C increase reduces cardiovascular outcomes in type 2 diabetes patients treated with up to 1.8 mg daily dose (109)

Status of approval in the U.S. and the European Union (EU), main mechanism of action, reported mean weight loss outcomes, and impact on parameters of metabolic health. Data are only included from randomized controlled trials with a duration of ≥52 weeks.

Abbreviations: BID, bis in die, twice a day; BP, blood pressure; ER, extended release; GLP-1, glucagon-like peptide-1; MHO, metabolically healthy obesity; QD, quaque die, once daily; SR, short release; TID, ter in die, three times a day. Adapted from references (102, 103); data on mean percentage of weight loss are from reference (102).

However, chronic, progredient, and relapsing diseases such as type 2 diabetes and obesity often require combination therapies. In addition to simply combining approved medications is that of using combination drugs (Table 2); collaborative research efforts by the laboratories of R. DiMarchi and M. Tschöp led to the discovery of several peptides with varying degrees of GLP-1 and glucagon coagonism (106, 112). Indeed, these and other coagonists derived from the proglucagon family are now advanced in the clinical development path (Table 3) and appear to be promising tools for the future pharmacotherapy of obesity. The first GLP-1/glucagon coagonist (MEDI0382) has already been studied in a Phase 2 clinical trial (113). Moreover, based on the metabolic benefits demonstrated for GLP-1/glucagon and GLP-1/GIP coagonists (“twincretins”), triagonists targeting all 3 incretin receptors have been systematically developed with even stronger efficacy compared to twincretins on weight loss and obesity-related traits, such as reducing liver fat (114, 115). These potential antiobesity pharmacotherapies of the future are part of a fast growing pipeline of drugs and targets for the urgently needed obesity pharmacotherapy (Table 3) (116). Most of these drugs are in preclinical development or at early stages of clinical development and include centrally acting agents (setmelanotide, neuropeptide Y antagonists, peptide YY, and cannabinoid type-1 receptor blockers) (117–120), amylin mimetics (davalintide, dual amylin, and calcitonin receptor agonists) (103, 116), leptin analogues (combination pramlintide-metreleptin) (120), FGF-21 (121), GDF-15 (122, 123), methionine aminopeptidase 2 inhibitor (beloranib), lipase inhibitors (cetilistat), triple monoamine reuptake inhibitor (tesofensine), antiobesity vaccines (ghrelin, somatostatin, and adenovirus 36) reviewed in reference 104), or synergistically targeting the cold nicotinic receptors (124).

**Table 3.** Examples of molecules or targets in development for obesity treatment.

Molecule or Class of Drugs	Mode of Action	Example Drugs and Status in Development	References
Semaglutide	GLP-1 receptor agonist	Approved for treatment of type 2 diabetes (1mg once weekly, sc injection), Phase 3 trials for obesity (2.4mg once weekly sc injections)	(110, 111)
Dual incretin agonists, “Twincretins”	GLP-1/glucagon coagonists, GLP-1/GIP coagonists	Phase 2 GLP-1/glucagon coagonists (e.g. MEDI0382) GLP-1/GIP coagonists (e.g. Tirzepatide, LY3298176; NNC9204-1177)	(104, 106, 112, 113)
Triagonists of the incretin system	GLP-1/ GIP/glucagon	Phase 1b (e.g. NNC9204-1706) preclinical	(114, 115)
Setmelanotide	MC4R-agonist target	Phase 2 (eg, RM-493)	(118, 119)
Amylin analogues	Amylin agonism	Phase 1–2 (eg, AM833; Davalintide: AC2307)	(103, 116)
PYY analogue	PYY agonism	Phase 1b (eg, PYY1562)	(103, 116, 117)
FGF21	Stimulation of glucose uptake, adiponectin secretion	Obesity: Phase 1b Type 2 diabetes: Phase 2	(121)
GDF-15	-	Preclinical	(122, 123)
Leptin analogues	Human recombinant leptin analogue	Phase Metreleptin (Myalept) and pramlintide-metreleptin combination	(121)
Velneperit	Neuropeptide Y5 receptor antagonist	Preclinical (eg, S-2367)	(103, 116)
Cannabinoid type-1 receptor blockers	Antagonism of cannabinoid type-1 receptors	Preclinical studies (eg, SR141716, AM251, AM 6545)	(103, 116)
Icilin/ dimethylphenyl-piperazinium (DMPP)	Activation of cold and nicotinic receptors	Preclinical studies	(124)

Adapted from references (103, 104, 105, 118).

Abbreviations: FGF-21, fibroblast growth factor-21; GDF-15, growth differentiation factor-15; sc, subcutaneous; GLP-1, glucagon-like peptide-1; PYY, peptide YY.

In summary, currently approved drugs and pharmacological obesity therapies in development have the potential to produce health improvements and convert MUO into MHO even without reaching a normal body weight.

## Conclusions

Metabolically healthy obesity is a concept derived from clinical observations that a subgroup of up to a third of people with obesity do not exhibit overt cardiometabolic abnormalities. Recently, standardized definitions of MHO have been proposed, which are relevant for clinical research about the differences in obesity-related morbidity and mortality between MHO and MUO. The risk to developing cardiometabolic diseases is lower in people with MHO compared to MUO. Whether MHO has additional implications for clinical obesity treatment remains uncertain, but individual treatment decisions should consider metabolic and cardiovascular abnormalities to reduce the risk for premature mortality, CVD, type 2 diabetes, and cancer in all patients with obesity.

The concept of MHO, as a human model system, can provide important insights to unravel the mechanisms of how fat accumulation, adverse fat distribution, and adipose tissue dysfunction may cause metabolic and cardiovascular abnormalities. In this context, the role of individual factors reflecting or causing MHO, including lower liver and visceral fat mass but higher leg fat content, greater cardiorespiratory fitness, and physical activity, insulin sensitivity, lower levels of inflammatory markers, and others need to be investigated.

MHO is a transient phenotype with a particularly high prevalence in premenopausal women and lower frequencies with increasing age, which can convert into and from MUO during the natural course of obesity and in response to obesity treatment. Importantly, timely treatment of obesity should also be recommended to individuals with MHO because their risk of developing cardiometabolic diseases is still higher than in metabolically healthy lean people.

Future research should take advantage of MHO as a model to understand how obesity, adipose tissue expansion, cellular composition, and dysfunction contribute to obesity-associated cardiometabolic diseases. Both in clinical practice and research, the definition of metabolic health needs to be harmonized. Further epidemiological studies may identify determinants and modifiable risk factors for the better prevention of conversions from MHO to MUO and cardiometabolic disease manifestations. In addition, genetic factors potentially contributing to MHO beyond expected effects of fat distribution, body composition, and subcutaneous adipose tissue expandability should be explored. Finally, a better understanding of whether and how different obesity treatment strategies, including pharmacotherapy, may cause distinct responses in individuals with MHO versus MUO could facilitate individual treatment decisions based on the MHO phenotype.

## Additional Information

***Disclosure Summary:*** M.B. received honoraria as a consultant and speaker from Amgen, AstraZeneca, Bayer, Boehringer-Ingelheim, Lilly, Novo Nordisk, Novartis, and Sanofi.
